# Immune micro-environment and drug analysis of peritoneal endometriosis based on epithelial-mesenchymal transition classification

**DOI:** 10.3389/fendo.2022.1035158

**Published:** 2022-11-29

**Authors:** Qingli Quan, Jiabao Wu, Meixing Yu, Jia Tang

**Affiliations:** ^1^ NHC Key Laboratory of Male Reproduction and Genetics, Guangdong Provincial Reproductive Science Institute (Guangdong Provincial Fertility Hospital), Guangzhou, China; ^2^ Guangzhou Women and Children’s Medical Center, Guangzhou Medical University, Guangzhou, China

**Keywords:** epithelial-mesenchymal transition, peritoneal endometriosis, immune micro-environment, diagnostic model, drug susceptibility analysis

## Abstract

**Background:**

Epithelial-mesenchymal transition (EMT) is a complex event that drives polar epithelial cells transform from adherent cells to motile mesenchymal cells, in which are involved immune cells and stroma cells. EMT plays crucial roles in migration and invasion of endometriosis. The interaction of endometrial implants with the surrounding peritoneal micro-environment probably affects the development of peritoneal endometriosis. To date, very few studies have been carried out on peritoneal endometriosis sub-type classification and micro-environment analysis based on EMT. The purpose of this study is to investigate the potential application of EMT-based classification in precise diagnosis and treatment of peritoneal endometriosis.

**Method:**

Based on EMT hallmark genes, 76 peritoneal endometriosis samples were classified into two clusters by consistent cluster classification. EMT scores, which calculated by Z score of 8 epithelial cell marker genes and 8 mesenchymal cell marker genes, were compared in two clusters. Then, immune scores and the abundances of corresponding immune cells, stroma scores and the abundances of corresponding stroma cells were analyzed by the “xCell” package. Futhermore, a diagnostic model was constructed based on 9 diagnostic markers which related to immune score and stroma score by Lasso-Logistic regression analysis. Finally, based on EMT classification, a total of 8 targeted drugs against two clusters were screened out by drug susceptibility analysis *via* “pRRophetic” package.

**Results:**

Hallmark epithelial-mesenchymal transition was the mainly enriched pathway of differentially expressed genes between peritoneal endometriosis tissues and endometrium tissues. Compared with cluster 2, EMT score and the abundances of most infiltrating stroma cell were significantly higher, while the abundances of most infiltrating immune cells were dramatically less. The diagnostic model could accurately distinguish cluster 1 from cluster 2. Pathway analysis showed drug candidates targeting cluster 1 mainly act on the IGF-1 signaling pathway, and drug candidates targeting cluster 2 mainly block the EGFR signaling pathway.

**Conclusion:**

In peritoneal endometriosis, EMT was probably promoted by stroma cell infiltration and inhibited by immune cell infiltration. Besides, our study highlighted the potential uses of the EMT classification in the precise diagnosis and treatment of peritoneal endometriosis.

## Introduction

Endometriosis is characterized by the presence of normal endometrium (like stroma and glands) abnormally invaded in body parts other than the uterine cavity, which shares many characteristics with malignant tumour (1) (2). Although ectopic endometrial tissue can be implanted in any parts of body, abdominal cavity is one of the most frequently locations that endometriotic tissue implanted into, leading to peritoneal endometriosis (1–4). Over the past decades, several systems have been proposed for endometriosis classification. The most widely accepted is American Society for Reproductive Medicine (rASRM) classification and the updated Enzian classification (Supplement to ASRM Classification) (5). However, the rASRM score has limitations in deep infiltrating endometriosis description and Enzian classification has not included peritoneal endometriosis classification (6), which is greatly limiting accurate diagnosis and treatment of peritoneal endometriosis.

Epithelial-mesenchymal transition (EMT) lead to the increased motility *via* rearrangements of cellular contact junctions, loss of cell adhesion, apicobasal polarity and epithelial cell morphology, thus promoting lesion metastasis (7, 8). In general, EMT of ectopic endometrial tissue is more active than that of eutopic endometrial tissue, which may be beneficial for migration and invasion of ectopic tissue (9). After endometrium attaches to peritoneum, endometrial epithelial cells also undergo EMT (10). Furthermore, the expressions of EMT induced transcription factors that may trigger EMT were significantly increased in deep endometriotic lesions than in eutopic endometrium (11, 12). These indicate EMT is a factor contributing to progression of endometriosis. Classification based on EMT hallmarks has been widely used in diseases sub-classify (13, 14), we supposed classification based on EMT also has a potential to be used on peritoneal endometriosis classification.

Immune micro-environment affects EMT (15, 16). Peritoneal endometriosis is markedly characterized by increased numbers of peritoneal macrophages and elevated concentrations of pro-inflammatory chemokines, which associated with endometriosis-related pain and infertility (17, 18). Macrophages induced EMT in pancreatic cancer cells (19). And inflammatory mediators in retrograde menstrual fluid probably contribute to ectopic endometrial EMT in the presence of peritoneal hypoxia (20). Besides, in superficial peritoneal endometriosis, the migration and infiltration of peritoneal endometriotic tissue were also associated with the formation and differentiation of stroma cells, such as myofibroblasts and smooth muscles (SM)-like cells (21). All these made us curious about the differences in immune cell infiltration and stroma cell infiltration of peritoneal endometriosis classified based on EMT classification.

Here, we classified peritoneal endometriosis into two clusters based on EMT hallmark genes by consistent cluster classification, which is suitable for diseases classification from the perspective of molecular (22, 23) Then, we compared the immune micro-environment and stroma cells infiltration of two clusters. What was more, based on EMT classification, we established a diagnostic model and screened potential drugs against different clusters. In conclusion, our study provided a potential strategy for peritoneal endometriosis diagnosis and treatment.

## Methods and materials

### Data collection

The RNA sequencing dataset of 76 peritoneal endometriosis tissues and 37 endometrium tissues was fetched from GSE141549. The clinical information all subjects was provided in **Supplementary Table 1**. Another RNA sequencing dataset that containing 11 peritoneal endometriosis tissues and 11 endometrium tissues was GSE5108. The single cell RNA-seq dataset (ScRNA-Seq) of 8 peritoneal endometriosis tissues was fetched from GSE179640. All the above datasets were downloaded from GEO DataSet. EMT hallmark genes were referred from the HALLMARK_EPITHELIAL_MESENCHYMAL_TRANSITION gene set in Molecular Signatures Database v7.5.1 (https://www.gsea-msigdb.org/gsea/msigdb/). The data of msigdb.v7.4.entrez.gmt was downloaded from Gene Set Enrichment Analysis website (https://www.gsea-msigdb.org/gsea/msigdb/).

### Gene set enrichment analysis

In order to explore potential mechanisms of EMT in peritoneal endometriosis, we performed gene set enrichment analysis (GSEA) on GSE141549 and GSE5108. Firstly, logFc values of all genes between peritoneal endometriosis tissues and endometrium tissue genes were obtained by”limma” package. Then, GSEA based on msigdb.v7.4.entrez.gmt by “clusterProfiler” package were performed (24). At last, the results was visualized by gseaplot2 of the “enrichplot” package (25).

### Consistent cluster analysis based on EMT

To classify peritoneal endometriosis, we performed consistent clustering analysis on GSE141549 based on the 200 EMT hallmark genes by using the “ConsensusClusterPlus” package (26). Samples were divided into two clusters according to the expression characteristics of EMT hallmark genes.

### Single cell RNA-seq data analysis

ScRNA-Seq analysis and visualization for GSE179640 were performed with “Seurat” package (version 4.1.1) (27, 28). Briefly, we removed low-quality cells with feature RNA< 500 or > 6000 and mitochondrial reads > 20%. Then, the top 2000 highly variable genes were selected after the gene expression normalization. After gene expression integration, cells were clustered and two-dimensional visualization was performed using uniform manifold approximation and projection (UMAP). Clusters were annotated based on the average gene expression of the following major cell types: fibroblasts (COL1A1, COL3A1, COL1A2), macrophages/monocytes (CD68, MS4A4A, MS4A7, CD14), endothelial cells (PECAM1, VWF), epithelial cells (EPCAM), mesenchymal cells (VIM), CD8+ T cells (PTPRC, CD2, CD3G, CD8A), CD4+ T cells (PTPRC, CD2, CD3G, CD4), dendritic cells (DC) (IL3RA, CLEC4C), mast cells (KIT, TPSB2, TPSAB1), natural killer cells (NK) (NCAM1) and neutrophils (FCGR3A) (29–32).

### EMT score calculation

To screen mesenchymal cell marker genes and epithelial marker cell genes for EMT score of peritoneal endometriosis, we firstly referenced 8 epithelial cell marker genes (CD24, CDH1, DSP, EPCAM, FOLR1, KRTI8, KRT19 and OCLN) and 14 mesenchymal cell marker genes (ACTA2, CD44, CDH2, FN1, ITGA5, MMP2, S100A4, SNAI2, TNC, TWIST1, VIM, WNT5A, ZEB1 and ZEB2) of the CellMarker website (http://xteam.xbio.top/CellMarker/). Then, we compared the expression of these genes in epithelial cells cluster and mesenchymal cells cluster (GSE179640). Finally, 8 epithelial genes and 8 mesenchymal genes were selected for EMT score. EMT score was the sum of Z scores of mesenchymal genes minus the sum of Z scores of epithelial genes (33).

### Calculation of immune score, stroma score, abundances of immune cells and stroma cells

“xCell” provides a novel method to infer immune and stromal cell types, immune score and stroma score based on genetic characteristics (34). Here, we used the “xCell” package to analyze the relative abundance of immune cells and stroma cells, immune score and stroma score in peritoneal endometriosis samples.

### Screening and functional enrichment analysis of differentially expressed genes

In order to figure out the functional differences of the differentially expressed genes (DEGs), the differential genes between cluster 1 and cluster 2 were screened by using of the “limma” package (adj. p. val< 0.05, |log FC| > 1) (35). Then, the DEGs were analyzed by Gene Ontology (GO) and Kyoto Encyclopedia of Genes and Genomes (KEGG) through the website (https://cn.string-db.org/). By setting FDR< 0.05, the significant terms were selected and visualized with the “ggplot2” package (36).

### Weighted gene co-expression network analysis

Weighted correlation network analysis (WGCNA) can be used for finding clusters (modules) of highly correlated genes, for summarizing such clusters using the module eigengene or an intramodular hub gene (37). To identify immune score or stroma score associated modules or genes, the “WGCNA” package was used to construct the co-expression network analysis of the mRNA expression matrix of DEGs. samples were clustered according to pearson’s correlation analysis and the outliers were removed. The soft thresholding parameter (β) was selected when the scale free topology model fit > 0.85. Afterward, the adjacency matrix was transformed into a topological overlap matrix (TOM) and genes were assigned to different gene modules according to dissimilarity matrix (1-TOM). Similar dynamic modules were merged when coefficient of dissimilarity< 0.2. Pearson correlation analysis was performed to identify the module with the strongest association with immune score and stroma score. The module eigengenes related to immune score or stroma score were selected with gene significance (GS) > 0.55 and module membership (MM) > 0.85, respectively.

### Lasso-logistics regression

We extracted expression matrix of immune score and stroma score related genes from GSE141549. Then, 76 samples in this expression matrix were randomly divided into training dataset and test dataset in a ratio of 1:1. In the training dataset, the Lasso-Logisitic regression analysis was performed based on the classification information of cluster 1 and cluster 2 using the “glmnet” package (38). The diagnostic markers were screen and a diagnostic model was built. Furthermore, the diagnostic model was validated in the test dataset. The ROC curves were plotted using the “ROCR” package and AUC value was calculated (39).

### Drug susceptibility analysis

The “pRRophetic” package was used to analyze the half maximal inhibitory concentration (IC50) of 251 drugs (40). Then, the drug candidates for cluster 1 or cluster 2 were screened by setting the adj. p. val<0.05.

### Statistics of data

All statistical analyses performed in our study were conducted in R studio (version 4.1.2). Comparisons of mRNA expression were analyzed by Wilcoxon test. All correlation analyses were performed by Pearson correlation analysis using the “corrplot” package (41). Differences were significant when P < 0.05.

## Results

### The classification based on the EMT hallmark genes

**Figure 1:**
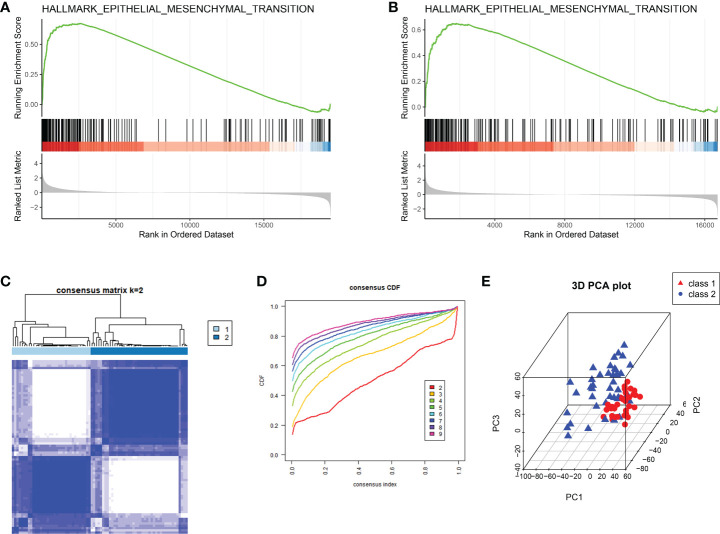
The classification of peritoneal endometriosis based on EMT. **(A, B)**. GSEA analysis of the whole transcriptome of GSE141549 and GSE5108, respectively. **(C)**. EMT modification patterns identified with K-means clustering. **(D)**. The cumulative distribution function (CDF) curve of the clustering. **(E)**. PCA plot of cluster1 and cluster2.

Results of GSEA on deferences gene expression between peritoneal endometriosis tissues and endometrium tissues of both GSE141549 and GSE5108 showed that hallmark epithelial- mesenchymal transition (EMT) was the mainly enriched pathway (**Figures 1A, B**). In order to analysis peritoneal endometriosis from the perspective of EMT, we performed consistent clustering analysis on GSE141549 that containing 76 patients with peritoneal endometriosis based on the EMT hallmark genes. Samples could be clearly divided into cluster 1 (n = 34) and cluster 2 (n = 42) (**Figures 1C-E**).

### EMT score comparison between cluster 1 and cluster 2

**Figure 2:**
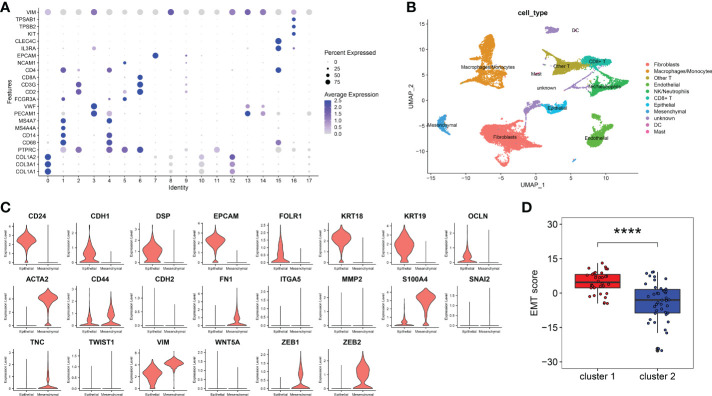
EMT score marker genes selection and EMT score calculation. **(A)**. Marker genes expression of 18 clusters were shown on bubble diagram. **(B)**. UMAP plots of 11 types of cells. Cells were colored for types. **(C)**. The expression level of EMT score marker genes in CellMarker website. The upper 8 genes were epithelial cell marker genes and the other 14 genes were mesenchymal cell marker genes. **(D)**. EMT score of cluster 1 and cluster 2. (p< 0.0001) (****p< 0.0001).

In order to calculate the EMT score of peritoneal endometriosis tissues, we performed ScRNA-Seq analysis on GSE179640 for selecting marker genes of epithelial cells and mesenchymal cells. The entire cell population was categorised into 18 major cell clusters. All cell clusters were identified as 11 cell types, consist of fbroblasts cells, macrophages/monocytes, endothelial cells, epithelial cells, other T cells, mesenchymal cells, CD8+ T cells, DC, mast cells, NK/neutrophils and unknown based on expression of markers (**Figures 2A, B**). We compared the expression of 8 epithelial cell marker genes and 14 mesenchymal cell marker genes in both epithelial cells and mesenchymal cells. Results showed the expression of 8 epithelial cell marker genes (CD24, CDH1, DSP, EPCAM, FOLR1, KRTI8, KRT19 and OCLN) were significantly higher in epithelial cells compared these with mesenchymal cell. And 8 mesenchymal cell marker genes (ACAT2, CD44, FN1, S1004A, TNC, VIM, ZEB1 and ZEB2) were just the oppose (**Figure 2C**). Hence, we selected these 16 genes as the marker genes for EMT score. Then, EMT score of peritoneal endometriosis (GSE141549) based on the Z score of these marker genes were calculated. Results showed that EMT score of cluster 1 was significantly higher than that of cluster 2 (p< 0.0001) (**Figure 2D**). Results indicated EMT appears much more robust in cluster 1 than that in cluster 2.

### Screening and functional enrichment analysis of the differential gene between cluster 1 and cluster 2.

**Figure 3:**
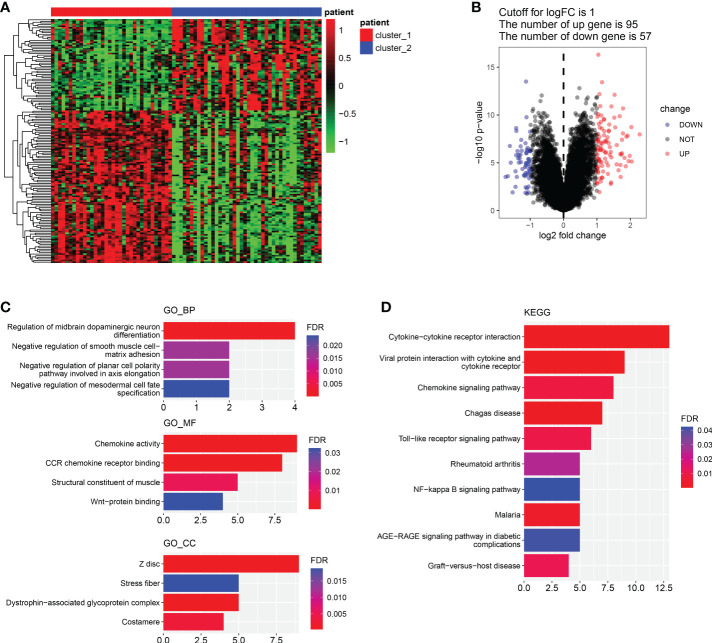
Differential genes screening and functional enrichment analysis. **(A, B)**. Heatmap of DEGs between cluster 1 and cluster 2. **(B)**. Volcano plot of DEGs between cluster 1 and cluster 2. **(C)**. GO enrichment analysis of DEGs about BP, MF and CC. **(D)**. KEGG-enriched analysis. (BP, biological process. MF, molecular function).

In order to explore the differences between cluster 1 and cluster 2 comprehensively, we analyzed the DEGs between cluster 1 and cluster 2. Results showed there were 95 up-regulated genes and 57 down-regulated genes in cluster 1 compared with cluster 2 (**Figures 3A, B**). Pathway enrichment indicated that the mainly enriched BP were Regulation of midbrain dopaminergic neuron differentiation and Negative regulation of smooth muscle cell matrix adhesion, the mainly enriched MF were Chemokine activity and CCR chemokine receptor binding, and the mainly CC were mainly Z disc, Stress fiber and Dystrophin-associated glycoprotein complex (**Figure 3C**). The mainly KEGG-enriched pathways were Cytokine-cytokine receptor interaction, Chemokine signaling, Toll-like receptor signaling pathway and NFkB signaling pathway (**Figure 3D**). Results showed DEGs between cluster 1 and cluster 2 mainly in volved in chemokines signaling pathways, including inflammatory chemokines pathways (Toll-like receptors pathway and NF-kappa B pathway).

### Screening of genes related to immune score and stroma score

**Figure 4:**
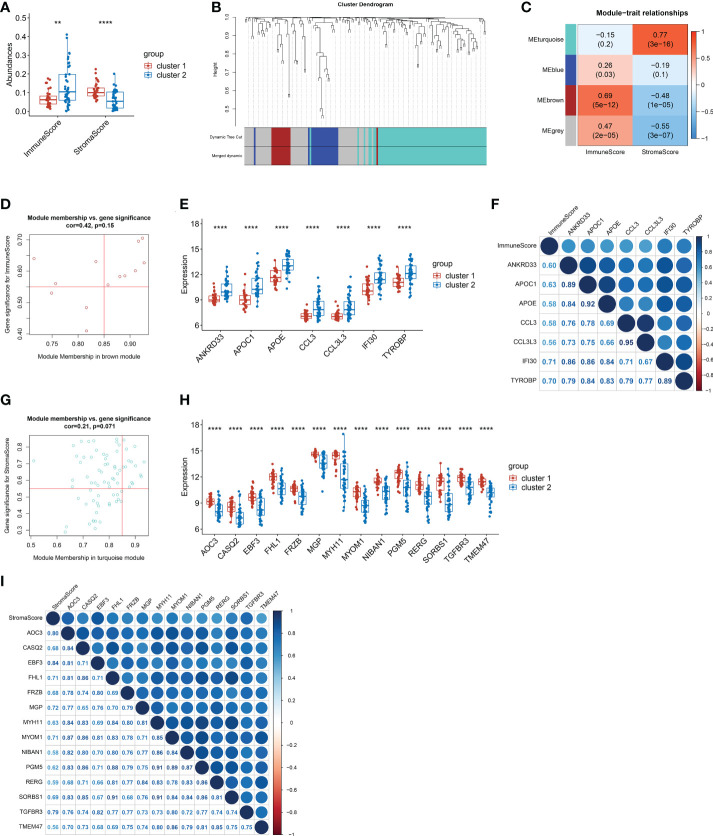
The screening of genes that related to Immune score and stroma score. **(A)**. The immune score and stroma score of cluster and cluster 2. **(B)**. Cluster dendrogram of the co-expression network modules. **(C)**. Correlations between the modules and immune scores, and correlations between the modules and immune scores (p-values were shown). **(D)**. Scatter plot analysis of the brown module. 7 Immune score-related genes were screened out in the upper-right area where GS > 0.55 and MM > 0.85. **(E)**. Comparison of the 7 immune score-related genes between cluster 1 and cluster 2. **(F)**. Correlation analysis between the 7 immune score-related genes. **(G)**. Scatter plot analysis of the turquoise module. 14 stroma score-related genes were screened out in the upper-right area where GS > 0.55 and MM > 0.85. **(H)**. Comparison of the 14 stroma score-related genes between cluster 1 and cluster 2. **(I)**. Correlation analysis between the 14 stroma score-related genes. (**p< 0.01; ****p< 0.0001; GS, gene significance. MM, module membership.).

Given that the functional differences between cluster 1 and cluster 2 were mainly enriched in chemotaxis and inflammatory responses, we further analyzed the immune micro-environment. The immune score of cluster 1 was significantly lower than that of cluster 2 (p<0.01), while the stroma score was dramatically higher than that of cluster 2 (p<0.0001) (**Figure 4A**). Furthermore, we selected immune score related gene and stroma score by WGCNA. Four modules were identified when the Diss Thres was set as 0.2 after merging dynamic modules, as shown in the clustering dendrograms (**Figure 4B**). The brown module and turquoise module were associated with Immune score and stroma score respectively (**Figure 4C**). Finally, 7 Immune score-related genes were set selected by setting GS>0.55 and MM>0.85 (**Figure 4D**). Results showed that the expression of all 7 Immune score-related genes in cluster 1 were significantly lower than those in cluster 2 (p<0.0001) (**Figure 4E**). All these 7 genes were significantly positively correlated with Immune score (p<0.0001) (**Figure 4F**). Similarly, 14 stroma score-related genes were selected and the expression of these 14 genes in cluster 1 were remarkably higher than those in cluster 2 (p<0.05) (**Figures 4G, H**). All 14-stroma score-related genes were significantly positively correlated with Immune score (p<0.05) (**Figure 4I**). In conclusion, the immune cells infiltration of cluster 1 was significantly higher than that of cluster 2, while the infiltration of stroma cells was remarkably lower in cluster 2. We speculated that, in peritoneal endometriosis lesions, high infiltration of immune cells inhibited the progression of EMT, while high infiltration of stroma cell contributes to EMT.

### The abundances of immune cells and stroma cells

**Figure 5:**
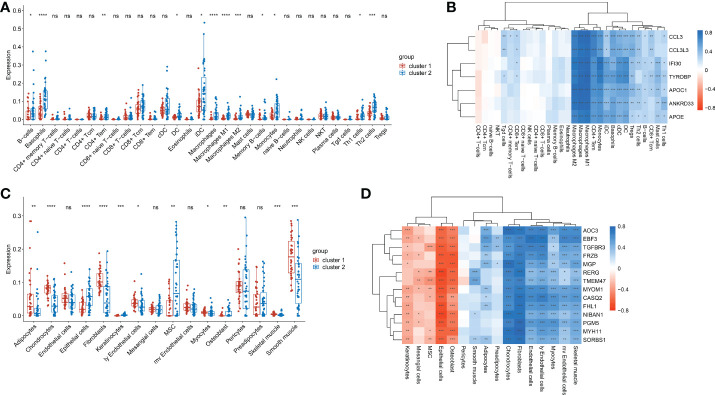
The abundances of immune cells and stroma cells in cluste1 and cluster2. **(A)**. Comparison of the immune cell abundances in cluster 1 and cluster2. **(B)**. Correlation of the immune cell abundances and the expression of immune-related genes. **(C)**. The comparison of the stroma cells abundances in cluster 1 and cluster2. **(D)**. Correlation of the stroma cells abundances and the expression of stroma score-related genes. (*p<0.05; **p<0.01; ***p<0.001; ****p<0.0001; ns, no significance).

Given the significant differences in immune score and stroma score between the two clusters, we further analyzed the abundances of immune cells and stroma cells. Results showed that the abundances of 12 kinds of immune cells, namely DC cells, iDC cells, Monocytes, Macrophages, M1 Macrophages, M2 Macrophages, Basophils, Th1 cells, Th2 cells, CD4+ Tem cells, B cells and memory B cells,were significantly lower in cluster 1 than that of cluster 2 (p< 0.05) (**Figure 5A**). Correlation analysis showed the abundances of above 12 kinds of immune cells were almost remarkably positively correlated with the expression of all 7 immune score-related genes (p< 0.05) (**Figure 5B**). This indicated that immune cells in cluster 2 were more active than those in cluster 1. And immune cells were positively regulated by immune score-related genes. Additionally, the abundance of Epithelial cells, Keratinocytes and Osteoblasts in cluster 1 were significantly lower than those in cluster 2 (p<0.05) (**Figure 5C**) and had significantly negative correlations with the stroma-related genes (**Figure 5D**). While the abundances of Fibroblasts, ly Endothelial cells, Myocytes, Chondrocytes and Skeletal muscle cells were significantly higher in cluster 1 than that of cluster 2 (p<0.05) (**Figure 5C**) had significantly positive correlations with the whole stroma-related genes (**Figure 5D**). In addition, abundances of Adipocytes and Smooth muscle cells were also higher in cluster 1. The epithelial cell abundance of cluster 1 was lower, which consistent with the EMT score (**Figure 2D**). It was suggested that the increased abundance of Fibroblasts, ly Endothelial cells, Skeletal muscle cells and Smooth muscle cells probably contribute to EMT in peritoneal endometriosis.

### Construction of the diagnostic model

**Figure 6:**
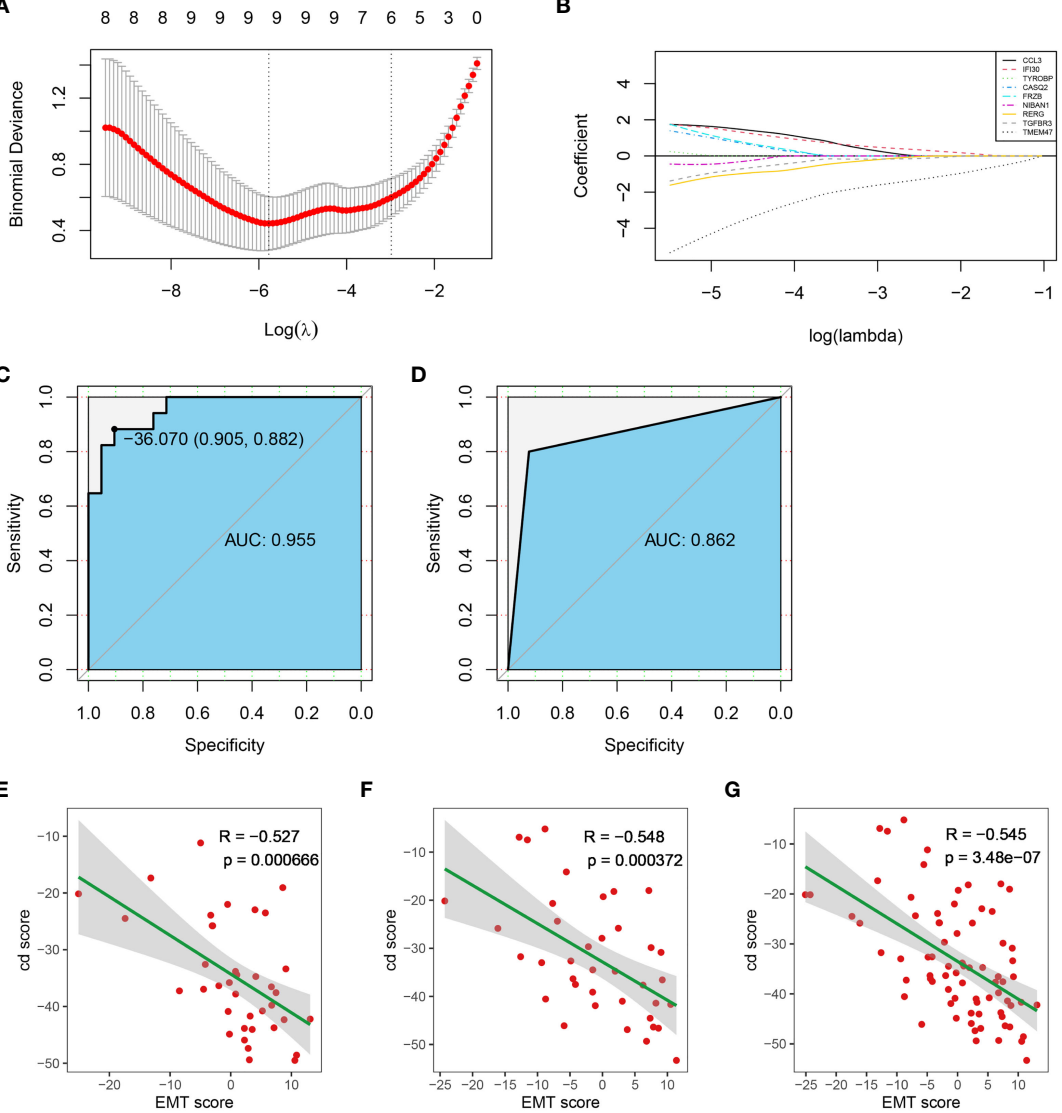
Construction of the diagnostic model. **(A)** Diagnostic markers screening. **(B)** The coefficients of all diagnostic markers. **(C)** ROC of the diagnostic model with the 14 diagnostic markers in the training dataset. **(D)** Validation of the diagnostic model in the test dataset. **(E–G)**. The correlation between cd score and EMT score in test dataset, training dataset and entire dataset, respectively. (AUC, Area Under Curve).


To construct a diagnostic model, diagnostic markers were screened from immune score- related genes and stroma score-related genes by lasso-logistic regression analysis in the training dataset. The minimum binomial deviance was obtained when log(λ) was -5.773583, and 9 genes were selected as diagnostic markers (**Figure 6A**). The coefficients of TMEM47 and FRZB were larger than the other 7 genes (**Figure 6B**) (**Supplementary Table 2**). A diagnostic model was constructed with the following formula:

$$cd\;score=\sum_{i=1}^{n}\left(Coefficient_{i}\times Expression_{i}\right)$$

The ROC analysis showed that the AUC of the training dataset was 0.955 when the cut-off value of the cd-score was -36.070 (**Figure 6C**). Sample was classified as cluster 1 when the cd-score was less than or equal to the cut-off value, otherwise sample was classified as cluster 2. According to the cut-off of the training dataset, the AUC of the test dataset was 0.862 (**Figure 6D**). Additionally, cd score was significantly negatively correlated with EMT score in training dataset, test dataset and entire dataset (**Figure 6E-G**). Therefore, the diagnostic model constructed from these 9 genes and their coefficients had high specificity and sensitivity.

### Candidate drug screening

**Figure 7:**
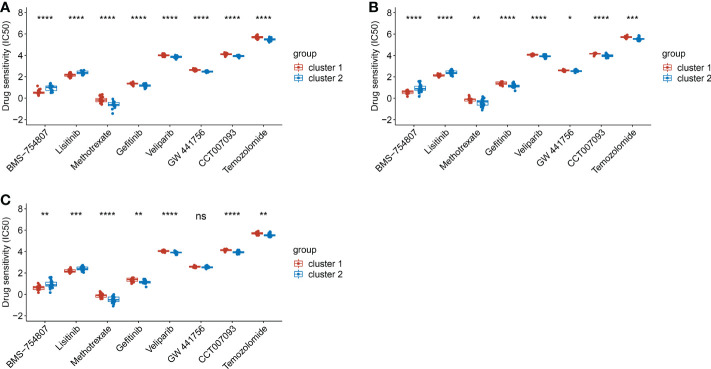
The comparison of drug sensitivity between cluster 1 and cluster 2. **(A)**. The comparison of drug sensitivity in training dataset. **(B)**. The comparison of drug sensitivity in test dataset. **(C)**. The comparison of drug sensitivity in the predicted cluster 1 and cluster 2 in test dataset. (*p< 0.05; **p< 0.01; ***p< 0.001; ****p< 0.0001).

Based on the clusters classified by EMT hallmark genes, drug susceptibility was analyzed. In the training dataset, the IC50 of BMS-754807 and Lisitinib in cluster 1 was significantly lower than that in cluster 2 (p<0.05), while the IC50 of Methotrexate, Gefitinib, Veliparib, GW 4441756, CCT007093 and Temozolomide in cluster 1 were remarkably higher in cluster 2 (p<0.0001) (**Figure 7A**). The drug susceptibility trends of all candidate drugs in the test dataset were consistent with that in the training dataset (**Figure 7B**). Then, we classified the dataset into cluster 1 and cluster 2 by the diagnostic model we established. Except for GW 441756, the susceptibility trends of all candidate drugs in the test dataset predicted by the above diagnostic model were also consistent with the training dataset. (**Figure 7C**). Results showed BMS-754807 and Lisitinib were more sensitive for cluster 1, while Methotrexate, Gefitinib, Veliparib, CCT007093 and Temozolomide were more sensitive for cluster 2. It was suggested that the diagnostic classification models we established can be used for drug screening.

## Discussion

Over decades, endometriosis classified traditionally based on lesion appearance, pelvic adhesions, or/and anatomic location of disease (42), but none of the current classification systems classify peritoneal endometriosis from molecular perspective. Here, we classified peritoneal endometriosis into two cluster based on EMT hallmark genes and found EMT scores of cluster 1 was significantly higher than cluster 2. What was more, we also found EMT in peritoneal endometriosis was related with both immune cell infiltration and stroma cell infiltration. In addition, based on immune score-related genes and stroma score-related genes, we established a diagnostic model and screened candidate drugs. Our study provided new ideas for classification, diagnosis and treatment of peritoneal endometriosis.

EMT is involved in the process of endometriosis. The migration and invasion abilities of endometrial stromal cells enhanced by facilitated EMT, and conversely inhibited EMT-related proteins reduced the volume and weight of endometriotic lesions in mice model (43–45). In pathological and physiological EMT, both stroma cells and immune cells are involved (46–48). Researches concerning stroma cell involve in EMT are not rare. Adipocytes promote EMT progression by reducing epithelial cell characteristics or inducing EMT-related phenotypes and thus promote tumor invasiveness (49, 50). Ly endothelial cells mediate the preferential migration of cells that undergoing EMT to lymphatic vessels by secreted pro-inflammatory cytokines (51). Chemokines promote pulmonary fibrosis by promoting EMT (52). EMT induced tissue fibrosis, which probably stimulate the production of fibroblasts in (53). Here we found not only the stroma score but also the abundances of most infiltrating stroma cells were significantly higher in cluster 1 than these in cluster 2, including fibroblasts, adipocytes, ly endothelial cells, chondrocytes, skeletal muscle cells and smooth muscle cells. We proposed that the infiltration of stroma cells probably contribute to EMT in peritoneal endometriosis. Besides, T and B cells, DC cells and tumor-associated macrophages that present in the tumor micro-environment induce EMT (54). Macrophages may induce pathological EMT of epithelial cells in a denomyosis (55). EMT is strongly associated with a highly immunosuppressive environment (15). We found the immune score was significantly lower in cluster 1 in than that in cluster 2, while the abundances of all infiltrating immune cells were significantly higher in cluster 2 than that in cluster 1, particularly macrophages, DC cells, CD4+T cells and B cells. Here, we proposed immune cell infiltration possibly inhibited the EMT of peritoneal endometriosis, especially macrophages, DC cells, CD4+T cells and B cells. Therefore, EMT classification is meaningful for peritoneal endometriosis accurate diagnosis and treatment.

Additionally, stroma score- and immune score-related genes possibably participate in stromal cells and immune cells infiltration. Aoc3 is an endothelial adhesion molecule that contributes to the extravasation of neutrophils, macrophages, and lymphocytes to sites of inflammation (56). CASQ2 is a calcium binding protein that stores calcium for muscle function (57). FRZB is involved in the regulation of chondrocytes development (58). MGP is a vitamin K-dependent protein, which is synthesized in bone and many other mesenchymal cells, which is also highly expressed by vascular smooth muscle cells (VSMCs) and chondrocytes (59). CCL3 and CCL3L3 are chemokines that produced by macrophage and monocyte respectively (60, 61). Ifi30 is an IFN-γ-inducible protein that is involved in MHC class II-restricted antigen processing and MHC class I-restricted cross-presentation pathways of adaptive immunity (62). Therefore, it was suggested that these genes regulate stroma cells and immune cellsinfiltration in peritoneal endometrisis.

To date, drugs treatment for endometriosis are mainly based on hormone regulation and inflammation inhibition, rarely concerning EMT. Here, based on EMT classification, we selected 2 candidate drugs for cluster 1 and 6 candidate drugs for cluster 2. As for cluster 2 drugs, Methotrexate blocks tumor cell proliferation mainly through the inhibition of dihydrofolate reductase (DHFR), which is also an immunosuppression (63). Gefitinib is a small molecule inhibitor of epidermal growth factor receptor (EGFR) tyrosine kinase (64). Veliparib is an inhibitor of PARP1 and PARP2 (65). GW 4441756 is a selective TrkA (NTRK1) inhibitor. CCT007093 is an inhibitor of protein phosphatase 1D (PPM1D Wip1) (66). Temozolomide reduces the proliferative activity of tumor cells (67). Pathway enrichment analysis found that drugs for cluster 2 mainly acted on the EGFR signaling pathway (**Supplementary Figure 1**). And restraining EGFR pathway can inhibit EMT progression (68, 69). Among drugs for cluster 1, BMS-754807 is a potent small molecule inhibitor of IGF-1R/IR family kinases. Lisitinib is a dual inhibitor of IGF-1 and insulin receptor (IR) (70). IGF-1 is expressed in ectopic endometrial stroma cells (71). In addition, IGF-1 concentration in peritoneal fluid of patients with endometriosis are significantly higher than that of normal controls (72, 73). On the other hand, the peritoneal mesothelial cells with insufficient IGF-1R expression had lower migration ability and higher adhesion ability (74). In addition, inhibitors of IGF-1R hinder the growth of ectopic lesions and reverses the pain behavior in mice model (71, 73). It was indicated that inhibition of insulin-like growth factor pathway was crucial for the treatment for cluster 1. Of course, drugs we screened needed to be further validated.

In conclusion, we classified peritoneal endometriosis based on EMT. Then, we constructed diagnostic models based on the screened genes and performed drug screening. This will provide a new strategy for the precise diagnosis and medicine of peritoneal endometriosis.

## Data availability statement

Publicly available datasets were analyzed in this study. This data can be found here: https://www.ncbi.nlm.nih.gov/geo/query/acc.cgi?acc=GSE141549.

## Ethics statement

Ethical review and approval was not required for the animal study because Our study is based on sequencing data downloaded from the GEO database.

## Author contributions

JT and JW collected the research data and checked the data analysis. MY directed data analysis. QQ analyzed the data and wrote the draft. All authors contributed to the article and approved the submitted version.

## Funding

We acknowledge the PhD workstation of Guangdong Provincial Reproductive Science Institute (Guangdong Provincial Fertility Hospital) for funding support (NO.: BS202201).

## Conflict of interest

The authors declare that the research was conducted in the absence of any commercial or financial relationships that could be construed as a potential conflict of interest.

## Publisher’s note

All claims expressed in this article are solely those of the authors and do not necessarily represent those of their affiliated organizations, or those of the publisher, the editors and the reviewers. Any product that may be evaluated in this article, or claim that may be made by its manufacturer, is not guaranteed or endorsed by the publisher.

## Supplementary material

Supplementary Figure 1 Pathway enrichment of targets of cluster 2 drugs.

Supplementary Table 1 Clinical information of the participants.

Supplementary Table 2 Gene coef.

Supplementary Data Sheet 1 R script.
