# Supplementary figures and images for: Immune micro-environment and drug analysis of peritoneal endometriosis based on epithelial-mesenchymal transition classification
Source: Front Endocrinol (Lausanne). 2022 Nov 29;13:1035158. doi: 10.3389/fendo.2022.1035158 (PMC9745086; doi:10.3389/fendo.2022.1035158)

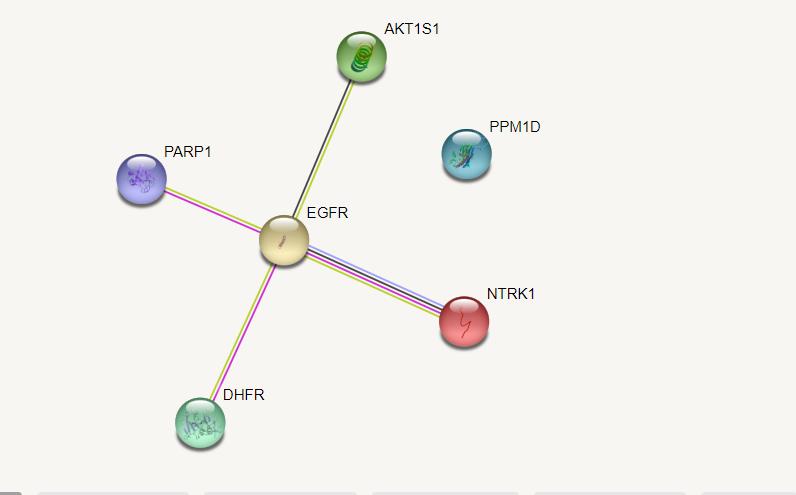

Supplement: Supplementary Figure 1 — Pathway enrichment of targets of cluster 2 drugs. [file Image_1.jpeg]
