# Supplementary material for: Immune micro-environment and drug analysis of peritoneal endometriosis based on epithelial-mesenchymal transition classification
Source: Front Endocrinol (Lausanne). 2022 Nov 29;13:1035158. doi: 10.3389/fendo.2022.1035158 (PMC9745086; doi:10.3389/fendo.2022.1035158)
Supplement: Supplementary Data Sheet 1 — R script. [file DataSheet_1.doc]

**###Consistent Cluster Analysis based on EMT**

rm(list = ls())

options(stringsAsFactors = F)

getwd()

setwd("E:/hall marker peri////")

library(ConsensusClusterPlus)

library(stringr)

library(dplyr)

library(survival)

library(survminer)

library(ggplot2)

library(ggpubr)

load("ex_ph2.Rdata")

hallmark<-read.csv("hallmark.csv",header=T)

table(ph2$Sample_description)

a1<-which(ph2$Sample_description=="Peritoneal endometriosis black lesion")

a2<-which(ph2$Sample_description=="Peritoneal endometriosis red lesion")

a3<-which(ph2$Sample_description=="Peritoneal endometriosis white lesion")

ph<-ph2[c(a1,a2,a3),]

w<-which(colnames(ex)%in%ph$Sample_geo_accession)

ex<-ex[,w]

gene<-hallmark

names(gene)[1]<-"Gene"

ex1<-ex[which(rownames(ex)%in%gene$Gene),]

setdiff(gene$Gene,rownames(ex1))

df<-as.matrix(ex1)

df <- sweep(df,1, apply(df,1,median,na.rm=T)) #

dim(df)

maxK <- 9

results <- ConsensusClusterPlus (df, maxK = maxK, reps = 1000, pItem = 0.8, pFeature = 1, clusterAlg = "pam",

distance="pearson", title="resultstrain_m5c", innerLinkage="complete", plot="png")

Kvec = 2:maxK

x1 = 0.1; x2 = 0.9

PAC = rep(NA,length(Kvec))

names(PAC) = paste("K=",Kvec,sep="")

for(i in Kvec){

M = results[[i]]$consensusMatrix

Fn = ecdf(M[lower.tri(M)])

PAC[i-1] = Fn(x2) - Fn(x1)

}

optK = Kvec[which.min(PAC)]

optK

icl = calcICL(results,

title="resultstrain_maxK_m5c0",

plot="pdf")

k2<-results[[2]]

class_k2<-as.data.frame(k2$consensusClass)

names(class_k2)[1]<-"class"

###########################################################################################################

**###DEG**

rm(list = ls())

options(stringsAsFactors = F)

getwd()

setwd("E://hall marker peri////")

load("ex_ph2.Rdata")

class<-read.csv("class_k2_m5c.csv",header = T)

class$class[which(class$class=="1")]<-"cluster 1"

class$class[which(class$class=="2")]<-"cluster 2"

table(class$class)

names(ph2)[1]<-"sample"

names(class)[1]<-"sample"

ph3<-merge(class,ph2,by="sample")

ex1<-merge(class,ex,by="sample")

ex2<-ex1[order(ex1$class,decreasing=F),]

colnames(ex2)[1:10]

table(ex2$class)

ex2$class[which(ex2$class=="cluster 1")]<-"cluster_1"

ex2$class[which(ex2$class=="cluster 2")]<-"cluster_2"

data<-ex2[,c(3:ncol(ex2))]

data1<-as.data.frame(t(data))

design <- model.matrix(~0+factor(group_list))

colnames(design)=levels(factor(group_list))

rownames(design)=colnames(data1)

library(limma)

table(group_list)

contrast.matrix<-makeContrasts(contrasts=c('cluster_1-cluster_2'),levels = design)

fit <- lmFit(data1,design)

fit1 <- contrasts.fit(fit, contrast.matrix)

fit2 <- eBayes(fit1)

class(fit2)

tT <- topTable(fit2, adjust="fdr", number=nrow(fit2))

tT2 <- topTable(fit2, number=nrow(fit2))

########################################

OAtT3<-tT2

OAtT3$Gene<-rownames(OAtT3)

logFC_cutoff <- with(OAtT3,mean(abs( logFC)) + 2*sd(abs( logFC)) )

logFC_cutoff = 1

mean(OAtT3$adj.P.Val)

fivenum((OAtT3$adj.P.Val))

mean(abs(OAtT3$logFC))

fivenum(( OAtT3$logFC))

colnames(OAtT3)

OAtT3$change = ifelse(OAtT3$adj.P.Val < 0.05 & abs(OAtT3$logFC) > logFC_cutoff, ifelse(OAtT3$logFC > logFC_cutoff ,'UP','DOWN'),'NOT')

this_tile <- paste0('Cutoff for logFC is ',round(logFC_cutoff,3),

'\nThe number of up gene is ',nrow(OAtT3[OAtT3$change =='UP',]) ,

'\nThe number of down gene is ',nrow(OAtT3[OAtT3$change =='DOWN',]))

table(OAtT3$change)

library(ggplot2)

g <- ggplot(data=OAtT3,

aes(x=logFC, y=-log10(P.Value), color=change)) +

geom_point(alpha=0.4, size=1.75) +

theme_set(theme_set(theme_bw(base_size=20)))+

xlab("log2 fold change") + ylab("-log10 p-value") +

ggtitle( this_tile ) +

theme(plot.title = element_text(size=15,hjust = 0.5))+

scale_colour_manual(values = c('blue','black','red'))+

annotate('text',x=OAtT3$logFC[OAtT3$logFC>40],y=-log10(OAtT3$P.Value[OAtT3$logFC>40]),

label=OAtT3$Gene[OAtT3$logFC>40])

g1<-g+theme_bw()+ theme(panel.grid.major = element_blank(),panel.grid.minor = element_blank())+

geom_vline(xintercept=0, linetype="dashed", color = "black",size=1)

print(g1)

ggsave(g1,filename = 'volcanoendo1.pdf',width = 4,height = 4)

data1$Gene=rownames(data1)

w<-which(OAtT3$change!="NOT")

OAtT4<-OAtT3[w,]

ex51<-merge(OAtT4,data1,by="Gene")

colnames(ex51)

rownames(ex51)<-ex51[,1]

colnames(ex51)

ex52<-ex51[,-c(1:8)]

data_matrix <-ex52

data_matrix=t(scale(t(data_matrix)))

fivenum(data_matrix)

data_matrix[data_matrix>1.2]=1.2

data_matrix[data_matrix< -1.2] = -1.2

library(pheatmap)

library(RColorBrewer)

bk = c(seq(-1.2,1.2, length=100)) #

annotation_col = data.frame(patient=group_list)

rownames(annotation_col)<-colnames(ex52)

ann_colors = list(patient = c(cluster_1= "red", cluster_2 = "blue"))

pheatmap(data_matrix,

breaks=bk,#

show_rownames = F,

annotation_col = annotation_col,

cluster_col = F,

border = NA,

cellheight = 1.8,

cellwidth = 4,

fontsize = 10,

fontsize_row = 5,

fontsize_col = 0.5,

annotation_colors = ann_colors,

color = colorRampPalette(c("green", "black", "red"))(100),

filename = 'pheatmap.png')

###########################################################################################

**###WGCNA**

rm(list = ls())

options(stringsAsFactors = F)

getwd()

setwd("E://hall marker peri////")

library(xCell)

xcell0<-xCellAnalysis(ex8)

xcell1<-as.data.frame(t(xcell0))

xcell1$sample<-rownames(xcell1)

xcell2<-merge(class,xcell1,by="sample")

xcell3<-xcell2[,c(1,67,68)]

#######

gene<-read.csv("OAtT5.csv")

w1<-which(gene$change!="NOT")

w2<-which(colnames(ex2)%in%gene[w1,]$Gene)

ex3<-ex2[,c(1,w2)]

colnames(xcell2)

xcell3<-xcell2[,c(1,67,68)]

data<-merge(xcell3,ex3,by="sample")

rownames(data)<-data$sample

data1<-data[,-c(1:3)]

pho<-data[,c(1:3)]

data2<-data1

library(WGCNA)

library(data.table)

library(stringr)

library(openxlsx)

exprSize = checkSets(data2,checkStructure = T)

gsg = goodSamplesGenes(data2)

gsg$allOK#

if (!gsg$allOK) {

data2 = data2[gsg$goodSamples, gsg$goodGenes]

}

gsg = goodSamplesGenes(data2)

gsg$allOK

nGenes = ncol(data2)

nSamples = nrow(data2)

save(data2,file='data2.Rdata')

powers = c(c(1:10), seq(from = 12, to=20, by=2))

sft = pickSoftThreshold(data2, powerVector = powers, verbose = 5)

pdf("001SFT.pdf",width=10,height = 6)

par(mfrow = c(1,2));

cex1 = 0.85;

plot(sft$fitIndices[,1], -sign(sft$fitIndices[,3])*sft$fitIndices[,2],

xlab="Soft Threshold (power)",ylab="Scale Free Topology Model Fit,signed R^2",type="n",

main = paste("Scale independence"));

text(sft$fitIndices[,1], -sign(sft$fitIndices[,3])*sft$fitIndices[,2],

labels=powers,cex=cex1,col="red");

abline(h=0.85,col="red")

plot(sft$fitIndices[,1], sft$fitIndices[,5],

xlab="Soft Threshold (power)",ylab="Mean Connectivity", type="n",

main = paste("Mean connectivity"))

text(sft$fitIndices[,1], sft$fitIndices[,5], labels=powers, cex=cex1,col="red")

dev.off()

softPower <- sft$powerEstimate

adjacency = adjacency(data2, power = softPower);

TOM = TOMsimilarity(adjacency);

dissTOM = 1-TOM

hierTOM = hclust(as.dist(dissTOM),method="average")

ADJ1_cor <- abs(WGCNA::cor( data2,use = "p" ))^softPower

k <- as.vector(apply(ADJ1_cor,2,sum,na.rm=T))

pdf("002K.pdf",width=10,height = 6)

par(mfrow = c(1,2))

hist(k)

scaleFreePlot(k,main="Check Scale free topology\n")

dev.off()

save(softPower,file='softPower.Rdata')

geneTree = hclust(as.dist(dissTOM), method = "average");

pdf("003Tree.pdf",width=10,height = 6)

plot(geneTree, xlab="", sub="", main = "Gene clustering on TOM-based dissimilarity",

labels = FALSE, hang = 0.04);

minModuleSize = 10;

dynamicMods = cutreeDynamic(dendro = geneTree, distM = dissTOM,

deepSplit = 2, pamRespectsDendro = FALSE,

minClusterSize = minModuleSize);

table(dynamicMods)

dynamicColors <- labels2colors(dynamicMods)

table(dynamicColors)

plotDendroAndColors(geneTree, dynamicColors, 'Dynamic Tree Cut',

dendroLabels = FALSE, addGuide = TRUE, hang = 0.03, guideHang = 0.05,

main = 'Gene dendrogram and module colors')

dev.off()

save(dynamicMods,file='dynamicMods.Rdata')

MEList = moduleEigengenes(data2, colors = dynamicColors)

MEs = MEList$eigengenes

MEDiss = 1-cor(MEs);

METree = hclust(as.dist(MEDiss), method = "average")

merge_modules = mergeCloseModules(data2, dynamicColors, cutHeight = MEDissThres, verbose = 3)

mergedColors = merge_modules$colors;

mergedMEs = merge_modules$newMEs;

pdf("007mergedTree.pdf",width=10,height = 6)

plotDendroAndColors(geneTree, cbind(dynamicColors, mergedColors),

c("Dynamic Tree Cut", "Merged dynamic"),

dendroLabels = FALSE, hang = 0.03,

addGuide = TRUE, guideHang = 0.05)

dev.off()

library(WGCNA)

library(data.table)

library(stringr)

library(openxlsx)

pho6<-pho1[,c(2:3)]

pdf("015Intramodular Connectivity and Module Membership000.pdf",width=6,height = 5)

moduleTraitCor_noFP <- cor(mergedMEs, pho6, use = "p");

moduleTraitPvalue_noFP = corPvalueStudent(moduleTraitCor_noFP, nSamples);

textMatrix_noFP <- paste(signif(moduleTraitCor_noFP, 2), "\n(", signif(moduleTraitPvalue_noFP, 1), ")", sep = "");

par(mar = c(6, 10, 3, 3));

labeledHeatmap(Matrix = moduleTraitCor_noFP,

xLabelsAngle = 45,

xLabelsAdj = 1,

xLabels = names(pho6),

yLabels = names(mergedMEs),

ySymbols = names(mergedMEs),

colorLabels = FALSE,

colors = blueWhiteRed(50),

textMatrix = textMatrix_noFP,

setStdMargins = FALSE,

cex.text = 1.2,

zlim = c(-1,1),

main = paste("Module-trait relationships"))

dev.off()

**#immunescore**

colnames(pho6)

x<-1

cor_ADR <- signif(WGCNA::cor(pho6,mergedMEs,use="p",method="pearson"),5)

p.values <- corPvalueStudent(cor_ADR,nSamples=nrow(pho6))

colnames(pho6)

colnames(cor_ADR)

Freq_MS_max_cor <- which.max(abs(cor_ADR[colnames(pho6)[x],-which(colnames(cor_ADR) == "MEgrey")]))

Freq_MS_max_p <- which.min(p.values[colnames(pho6)[x],-which(colnames(p.values) == "MEgrey")])

scolor<-Freq_MS_max_cor

ADJ1=abs(cor(data2,use="p"))^softPower

Alldegrees1=intramodularConnectivity(ADJ1, moduleColors)

datKME=signedKME(data2, mergedMEs, outputColumnName="MM.")

save(datKME,file = "datKME.Rdata")

ImmuneScore = as.data.frame(pho6[,x]);

names(ImmuneScore) = colnames(pho6)[x]

MET = orderMEs(cbind(mergedMEs, ImmuneScore))

corType="pearson"

if (corType=="pearson") {

geneModuleMembership = as.data.frame(cor(data2, mergedMEs, use = "p"))

MMPvalue = as.data.frame(corPvalueStudent(

as.matrix(geneModuleMembership), nSamples))

} else {

geneModuleMembershipA = bicorAndPvalue(data2, mergedMEs, robustY=ifelse(corType=="pearson",T,F))

geneModuleMembership = geneModuleMembershipA$bicor

MMPvalue = geneModuleMembershipA$p

}

if (corType=="pearson") {

geneTraitCor = as.data.frame(cor(data2, pho6, use = "p"))

geneTraitP = as.data.frame(corPvalueStudent(

as.matrix(geneTraitCor), nSamples))

} else {

geneTraitCorA = bicorAndPvalue(data2, pho6, robustY=ifelse(corType=="pearson",T,F))

geneTraitCor = as.data.frame(geneTraitCorA$bicor)

geneTraitP = as.data.frame(geneTraitCorA$p)

}

module = scolor

pheno = colnames(pho6)[x]

modNames = substring(colnames(mergedMEs), 3)

module_column = match(module, modNames)

pheno_column = match(pheno,colnames(pho6))

moduleGenes = moduleColors == module

pdf("019MMvsGS Immunescore.pdf",width=5,height = 5)

par(mfrow = c(1,1))

verboseScatterplot(abs(geneModuleMembership[moduleGenes, module_column]),

abs(geneTraitCor[moduleGenes, pheno_column]),

xlab = paste("Module Membership in", module, "module"),

ylab = paste("Gene significance for", pheno),

main = paste("Module membership vs. gene significance\n"),

cex.main = 1.2, cex.lab = 1.2, cex.axis = 1.2, col = module)

abline(h=0.55, col = "red")

abline(v=0.85, col = "red")

dev.off()

paste("MM.",scolor,sep = "")

wsc<-which(str_detect(colnames(datKME),paste("MM.",scolor,sep = "")))

head(data2[1:4,1:4])

class(data2)

FilterGenes= which(mergedColors==scolor)[abs(GS1)[which(mergedColors==scolor)]> 0.55 & abs(datKME[moduleGenes,wsc])>0.85]

trait_hubGenes<-colnames(data2)[FilterGenes]

write.csv(trait_hubGenes,"hubgene Immunescore.csv")

#############

#Stromascore

colnames(pho6)

x<-1

cor_ADR <- signif(WGCNA::cor(pho6,mergedMEs,use="p",method="pearson"),5)

p.values <- corPvalueStudent(cor_ADR,nSamples=nrow(pho6))

colnames(pho6)

colnames(cor_ADR)

Freq_MS_max_cor <- which.max(abs(cor_ADR[colnames(pho6)[x],-which(colnames(cor_ADR) == "MEgrey")]))

Freq_MS_max_p <- which.min(p.values[colnames(pho6)[x],-which(colnames(p.values) == "MEgrey")])

scolor<-Freq_MS_max_cor

ADJ1=abs(cor(data2,use="p"))^softPower

Alldegrees1=intramodularConnectivity(ADJ1, moduleColors)

datKME=signedKME(data2, mergedMEs, outputColumnName="MM.")

save(datKME,file = "datKME.Rdata")

ImmuneScore = as.data.frame(pho6[,x]);

names(ImmuneScore) = colnames(pho6)[x]

MET = orderMEs(cbind(mergedMEs, ImmuneScore))

corType="pearson"

if (corType=="pearson") {

geneModuleMembership = as.data.frame(cor(data2, mergedMEs, use = "p"))

MMPvalue = as.data.frame(corPvalueStudent(

as.matrix(geneModuleMembership), nSamples))

} else {

geneModuleMembershipA = bicorAndPvalue(data2, mergedMEs, robustY=ifelse(corType=="pearson",T,F))

geneModuleMembership = geneModuleMembershipA$bicor

MMPvalue = geneModuleMembershipA$p

}

if (corType=="pearson") {

geneTraitCor = as.data.frame(cor(data2, pho6, use = "p"))

geneTraitP = as.data.frame(corPvalueStudent(

as.matrix(geneTraitCor), nSamples))

} else {

geneTraitCorA = bicorAndPvalue(data2, pho6, robustY=ifelse(corType=="pearson",T,F))

geneTraitCor = as.data.frame(geneTraitCorA$bicor)

geneTraitP = as.data.frame(geneTraitCorA$p)

}

module = scolor

pheno = colnames(pho6)[x]

modNames = substring(colnames(mergedMEs), 3)

module_column = match(module, modNames)

pheno_column = match(pheno,colnames(pho6))

moduleGenes = moduleColors == module

pdf("019MMvsGS Immunescore.pdf",width=5,height = 5)

par(mfrow = c(1,1))

verboseScatterplot(abs(geneModuleMembership[moduleGenes, module_column]),

abs(geneTraitCor[moduleGenes, pheno_column]),

xlab = paste("Module Membership in", module, "module"),

ylab = paste("Gene significance for", pheno),

main = paste("Module membership vs. gene significance\n"),

cex.main = 1.2, cex.lab = 1.2, cex.axis = 1.2, col = module)

abline(h=0.55, col = "red")

abline(v=0.85, col = "red")

dev.off()

paste("MM.",scolor,sep = "")

wsc<-which(str_detect(colnames(datKME),paste("MM.",scolor,sep = "")))

head(data2[1:4,1:4])

class(data2)

FilterGenes= which(mergedColors==scolor)[abs(GS1)[which(mergedColors==scolor)]> 0.55 & abs(datKME[moduleGenes,wsc])>0.85]

trait_hubGenes<-colnames(data2)[FilterGenes]

write.csv(trait_hubGenes,"hubgene stromascore.csv")

##############################################################################################

**###Correlation**

rm(list = ls())

options(stringsAsFactors = F)

getwd()

setwd("E://hall marker peri/")

library(PerformanceAnalytics)

library(zoo)

library(corrplot)

library(lattice)

library(Formula)

library(ggplot2)

library(pheatmap)

library(RColorBrewer)

load("ex2.Rdata")

load("xcell2.Rdata")

**#**Immunescore

keygene<-read.csv("hubgene Immunescore.csv",header = T)

w2<-which(colnames(ex2)%in%keygene$x)

colnames(ex2)[1:10]

ex3<-ex2[,c(1,2,w2)]

colnames(ex3)[1:10]

colnames(ex3)

colnames(xcell2)

xcell3<-xcell2[,c(1,67)]

ex4<-merge(xcell3,ex3,by="sample")

s1<-c(1,3)

exgenallimgs3<-ex4[,-s1]

exoas2 <- rcorr(as.matrix(exgenallimgs3),type = "pearson")

pdf("xiangguanxing_keygene Immunescore.pdf",width=5.8,height = 5)

corrplot(exoas2$r, method = "circle", type = "upper", tl.pos = "lt",tl.cex = 1,tl.col="black", tl.srt=45)

corrplot(exoas2$r, add = TRUE, type = "lower", method = "number", diag = FALSE,

tl.pos = "n", cl.pos = "n",number.cex=1.2)

dev.off()

celltype<-read.csv("celltype.csv",header = T)

table(celltype$immune_stromal)

immunecell<-celltype[which(celltype$immune_stromal=="immune cell"),]$cell

ximmunecell<-xcell2[,c(1,2,which(colnames(xcell2)%in%immunecell))]

ximmunecell$class[which(ximmunecell$class=="1")]<-"cluster 1"

ximmunecell$class[which(ximmunecell$class=="2")]<-"cluster 2"

eximmune<-merge(ex3,ximmunecell,by="sample")

colnames(eximmune)

eximmune1<-eximmune[,c(3:9,11:ncol(eximmune))]

exgenallimgs3<-eximmune1

exoas2 <- rcorr(as.matrix(exgenallimgs3),type = "pearson")

r<-exoas2$r

p<-exoas2$P

colnames(r)

r2<-r[c(1:7),c(8:ncol(r))]

p2<-p[c(1:7),c(8:ncol(p))]

p21<-p2

if (!is.null(p21)){

sssmt <- p21<= 0.001

p21[sssmt] <-'***'

ssmt <- p21 >0.001& p21<= 0.01

p21[ssmt] <-'**'

smt <- p21 >0.01& p21 <=0.05

p21[smt] <- '*'

p21[!sssmt&!ssmt&!smt]<- ''

} else {

p21 <- F

}

data_matrix<-r2

max(r2)

min(r2)

bk = c(seq(-0.85,0.85, length=100))

pheatmap(data_matrix,

breaks=bk,#

legend_breaks=c(-0.8,-0.4,0,0.4,0.8),

show_rownames = T,

show_colnames = T,

cluster_col = T,

cluster_row = T,

border = NA,

cellheight = 25,

cellwidth = 12,

fontsize = 10,

fontsize_row = 10,

fontsize_col = 10,

display_numbers = p21,

color = colorRampPalette(c("#FF3300", "white","#0066CC"))(100),

filename = 'xiangguanxingp_keygene_immune_cell.pdf',width = 10,height = 8)

**#StromaScore**

keygene<-read.csv("hubgene StromaScore.csv",header = T)

w2<-which(colnames(ex2)%in%keygene$x)

colnames(ex2)[1:10]

ex3<-ex2[,c(1,2,w2)]

xcell3<-xcell2[,c(1,68)]

ex4<-merge(xcell3,ex3,by="sample")

colnames(ex4)

s1<-c(1,3)

exgenallimgs3<-ex4[,-s1]

exoas2 <- rcorr(as.matrix(exgenallimgs3),type = "pearson")

pdf("xiangguanxing_keygene StromaScore.pdf",width=7.8,height = 7)

corrplot(exoas2$r, method = "circle", type = "upper", tl.pos = "lt",tl.cex = 0.9,tl.col="black", tl.srt=45)

corrplot(exoas2$r, add = TRUE, type = "lower", method = "number", diag = FALSE,

tl.pos = "n", cl.pos = "n",number.cex=0.95)

dev.off()

celltype<-read.csv("celltype.csv",header = T)

table(celltype$immune_stromal)

stromacell<-celltype[which(celltype$immune_stromal=="stromal cell"),]$cell

xstromacell<-xcell2[,c(1,2,which(colnames(xcell2)%in%stromacell))]

xstromacell$class[which(xstromacell$class=="1")]<-"cluster 1"

xstromacell$class[which(xstromacell$class=="2")]<-"cluster 2"

names(xstromacell)

exstroma<-merge(ex3,xstromacell,by="sample")

colnames(exstroma)

exstroma1<-exstroma[,c(3:16,18:ncol(eximmune))]

exgenallimgs3<-exstroma1

exoas2 <- rcorr(as.matrix(exgenallimgs3),type = "pearson")

r<-exoas2$r

p<-exoas2$P

colnames(r)

r2<-r[c(1:14),c(15:ncol(r))]

p2<-p[c(1:14),c(15:ncol(p))]

p21<-p2

if (!is.null(p21)){

sssmt <- p21<= 0.001

p21[sssmt] <-'***'

ssmt <- p21 >0.001& p21<= 0.01

p21[ssmt] <-'**'

smt <- p21 >0.01& p21 <=0.05

p21[smt] <- '*'

p21[!sssmt&!ssmt&!smt]<- ''

} else {

p21 <- F

}

data_matrix<-r2

max(r2)

min(r2)

bk = c(seq(-0.8,0.8, length=100))

pheatmap(data_matrix,

breaks=bk,#

legend_breaks=c(-0.8,-0.4,0,0.4,0.8),

show_rownames = T,

show_colnames = T,

cluster_col = T,

cluster_row = T,

border = NA,

cellheight = 15,

cellwidth = 20,

fontsize = 10,

fontsize_row = 10,

fontsize_col = 10,

display_numbers = p21,

color = colorRampPalette(c("#FF3300", "white","#0066CC"))(100),

filename = 'xiangguanxingp_keygene_stroma_cell.pdf',width = 10,height = 8)

##################################################################################

**###Lasso-Logisitic Regression**

rm(list = ls())

options(stringsAsFactors = F)

getwd()

setwd("E:/hall marker peri//")

load("ex2.Rdata")

load("trait_hubGenes Immunescore.Rdata")

gene1<-trait_hubGenes

load("trait_hubGenes StromaScore.Rdata")

gene2<-trait_hubGenes

w1<-which(colnames(ex2)%in%gene1)

w2<-which(colnames(ex2)%in%gene2)

ex3<-ex2[,c(2,w1,w2)]

data<-ex3

colnames(data) <- gsub("-", ".", colnames(data), fixed = TRUE)

table(data$class)

levels(factor(data$class))

data$class=as.factor(data$class)

data$class=as.numeric(data$class)

sum(is.na(data))

library(sampling)

train_id <- strata(data, "class", size = rev(round(table(data$class) * 0.5)))$ID_unit

train_data <- data[train_id, ]

test_data <- data[-train_id, ]

prop.table(table(train_data$class))

table(test_data$class)

library(stringi)

library(Rcpp)

library(ElemStatLearn) #contains the data

library(car) #package to calculate Variance Inflation Factor

library(corrplot) #correlation plots

library(leaps) #best subsets regression

library(glmnet) #allows ridge regression, LASSO and elastic net

library(caret)

x <- as.matrix(train_data[, -c(1)])

y <- train_data[, c(1)]

fit <- glmnet(x, y, family = "binomial", alpha = 1)

print(fit)

set.seed(1)

fit_cv <- cv.glmnet(x, y, family = "binomial", alpha = 1,

type.measure = "auc")

pdf("00lasso01.pdf",width=5,height = 3.8)

plot(fit_cv)

dev.off()

log(fit_cv$lambda.min)

library(data.table)

get_coe<-function(the_fit,the_lamb){

Coefficients <- coef(the_fit,s=the_lamb)

Active.Index <- which(Coefficients !=0)

Active.Coefficients <- Coefficients[Active.Index]

re <- data.frame(rownames(Coefficients)[Active.Index],Active.Coefficients)

re <- data.table("var_names"=rownames(Coefficients)[Active.Index],

"coef"=Active.Coefficients)

re$expcoef <- exp(re$coef)

return(re[order(expcoef)])

}

get_coe(fit_cv,fit_cv$lambda.min)

gene_coef<-get_coe(fit_cv,fit_cv$lambda.min)

write.csv(gene_coef,"gene_coef.csv")

############################################################

get_plot<- function(the_fit,the_fit_cv,the_lamb,toplot = seq(1,50,2)){

Coefficients <- coef(the_fit, s = the_lamb)

Active.Index <- which(Coefficients != 0)

coeall <- coef(the_fit, s = the_fit_cv$lambda[toplot])

coe <- coeall[Active.Index[-1],]

ylims=c(-max(abs(coe)),max(abs(coe)))

sp <- spline(log(the_fit_cv$lambda[toplot]),coe[1,],n=100)

plot(sp,type='l',col =1,lty=1,

ylim = ylims,ylab = 'Coefficient', xlab = 'log(lambda)')

abline(h=0)

for(i in c(2:nrow(coe))){

lines(spline(log(the_fit_cv$lambda[toplot]),coe[i,],n=1000),

col =i,lty=i)

}

legend("topright",legend=rownames(coe),col=c(1:nrow(coe)),

lty=c(1:nrow(coe)),

cex=0.42)

}

pdf("00lasso02.pdf",width=5,height = 3.8)

get_plot(fit,fit_cv,exp(log(fit_cv$lambda.min)))

dev.off()

##################################################################

w3<-which(colnames(train_data)%in%gene_coef$var_names)

train01<-train_data[,c(w3,1)]

gene01<-gene_coef[order(gene_coef$var_names,decreasing=F),]

gene02<-gene01[-1,]

gene02<-as.data.frame(gene02)

gene02$var_names

train01$score<-train01[,1]*gene02[1,2]+train01[,2]*gene02[2,2]+train01[,3]*gene02[3,2]+

train01[,4]*gene02[4,2]+train01[,5]*gene02[5,2]+train01[,6]*gene02[6,2]+

train01[,7]*gene02[7,2]+train01[,8]*gene02[8,2]+train01[,9]*gene02[9,2]

train02<-train01[order(train01$score,decreasing=F),]

modelroc <- roc(train02$class,train02$score)

pdf("00lasso_ROC_train00.pdf",width=4,height = 4)

plot(modelroc, print.auc=TRUE, auc.polygon=TRUE, grid=c(0.1, 0.2),

grid.col=c("green", "red"), max.auc.polygon=TRUE,

auc.polygon.col="skyblue", print.thres=TRUE)

dev.off()

w3<-which(colnames(test_data)%in%gene_coef$var_names)

test01<-test_data[,c(w3,1)]

colnames(test01)

gene01<-gene_coef[order(gene_coef$var_names,decreasing=F),]

gene02<-gene01[-1,]

gene02<-as.data.frame(gene02)

gene02$var_names

test01$score<-test01[,1]*gene02[1,2]+test01[,2]*gene02[2,2]+test01[,3]*gene02[3,2]+

test01[,4]*gene02[4,2]+test01[,5]*gene02[5,2]+test01[,6]*gene02[6,2]+

test01[,7]*gene02[7,2]+test01[,8]*gene02[8,2]+test01[,9]*gene02[9,2]

test02<-test01[order(test01$score,decreasing=F),]

test02$pred<-ifelse(test02$score>-36.070,'2','1')

test02$pred<-as.factor(test02$pred)

test02$pred<-as.numeric(test02$pred)

modelroct <- roc(test02$class,test02$pred)

pdf("00lasso_ROC_test00.pdf",width=4,height = 4)

plot(modelroct, print.auc=TRUE, auc.polygon=TRUE, grid=c(0.1, 0.2),

grid.col=c("green", "red"), max.auc.polygon=TRUE,

auc.polygon.col="skyblue", #print.thres=TRUE

)

dev.off()

########################################################################################

**###IC50**

rm(list = ls())

options(stringsAsFactors = F)

getwd()

setwd("E:/hall marker peri//")

load("ex_ph.Rdata")

library(pRRophetic)

data<-as.matrix(ex)

library(parallel)

library(pRRophetic)

library(ggplot2)

data(PANCANCER_IC_Tue_Aug_9_15_28_57_2016)

data(cgp2016ExprRma)

possibleDrugs2016 <- unique( drugData2016$Drug.name)

possibleDrugs2016<-possibleDrugs2016

possibleDrugs2016 <- unique( drugData2016$Drug.name)

possibleDrugs2016<-possibleDrugs2016

system.time({

cl <- makeCluster(8)

results <- parLapply(cl,possibleDrugs2016,

function(x){

library(pRRophetic)

load("data_drug.Rdata")#

predictedPtype=pRRopheticPredict(

testMatrix=data,

drug=x,

tissueType = "all",

batchCorrect = "eb",

selection=1,

dataset = "cgp2016")

return(predictedPtype)

}) # lapply

stopCluster(cl)

})

IC50_result <- do.call('rbind',results)

rownames(IC50_result)<-possibleDrugs2016

IC501<-as.data.frame(t(IC50_result))

IC501$sample<-rownames(IC501)

colnames(train03)

ex6<-merge(train03[,c(1,(ncol(train03)-1))],IC501,by="sample")

ex6$class[which(ex6$class=="1")]<-"cluster 1"

ex6$class[which(ex6$class=="2")]<-"cluster 2"

library(tibble)

library(cowplot)

library(tidyverse)

library(ggplot2)

library(ggsci)

library(ggpubr)

library(dplyr)

colnames(ex6)

a1<-ex6

names(a1)[2]<-"group"

colnames(a1)

mydata<-a1 %>%

gather(key="gene",value="Expression", c(colnames(a1)[3:ncol(a1)])) %>%

dplyr::select(sample,gene,Expression,everything())

head(mydata)

table(mydata$group)

mydata$group = factor(mydata$group, levels=c('cluster 1','cluster 2'))

compare_means(Expression ~ group, data = mydata)

my_comparisons <- list( c("cluster 1", "cluster 2"))

options(scipen = 200)

p<-compare_means(Expression ~ group, data = mydata,group.by = "gene")

durg<-p[which(p$p.adj<0.05),]$gene

write.csv(p,"durg_train.csv")
